# Alcohol Induced Wernicke Encephalopathy with Atypical MRI Findings

**DOI:** 10.7759/cureus.5203

**Published:** 2019-07-22

**Authors:** Devansh Pandey, James L Kuhn, Hilda Tejero, James S Banks

**Affiliations:** 1 Miscellaneous, College of Osteopathic Medicine, Nova Southeastern University, Fort Lauderdale, USA; 2 Internal Medicine, Aventura Hospital and Medical Center, Aventura, USA; 3 Radiology, Aventura Hospital and Medical Center, Aventura, USA

**Keywords:** wernicke, wernicke-korsakoff, encephalopathy, neurology, neuroradiology, thiamine deficiency, thiamine deficiency, mri, alcoholism

## Abstract

Wernicke encephalopathy is a neurological complication of thiamine deficiency, usually in the setting of poor diet, classically with alcoholism. Patients present with acute onset of encephalopathy, oculomotor dysfunction, gait ataxia and memory impairment. If untreated, the disorder can result in severe morbidity and possibly death; patient outcomes are entirely dependent on prompt diagnosis and administration of parenteral thiamine. Although diagnosed clinically, the radiologist may be able to alert the referring clinician to the possibility of the disease when imaging features are observed, thereby improving the chance of treatment success. Although various imaging features have been ascribed to alcohol and non-alcohol related forms of Wernicke encephalopathy, recent literature suggests that such a distinction is not reliable, and that the causes of Wernicke encephalopathy are not readily distinguishable on MRI, as in the index case presented here.

## Introduction

Wernicke encephalopathy is a neurological complication of thiamine deficiency. It is usually caused by a poor diet accompanied by alcoholism [1-3]. Acute onset of encephalopathy, oculomotor dysfunction, and gait ataxia is the classic triad for diagnosis. However, patients typically present with just one or two of these symptoms [1, 4]. If left untreated, the disorder can result in severe morbidity and possibly death. Thus, patient outcomes depend entirely on prompt diagnosis and administration of parenteral thiamine [2, 5]. A majority of cases of Wernicke encephalopathy are diagnosed clinically. However, the radiologist may be able to alert the referring clinician to the possibility of the disease when particular imaging features are observed, thereby improving clinical outcomes. Although various imaging features have been ascribed to alcohol- and non-alcohol-related forms of Wernicke encephalopathy [3, 6], recent literature suggests that such a distinction is not reliable, and that the causes of Wernicke encephalopathy are not readily distinguishable on an MRI, as demonstrated by the index case presented here [7].

## Case presentation

A 45-year-old female presented after four days of nausea, vomiting, dizziness and falls. She had a history of alcohol abuse and admitted to currently drinking 1-2 bottles of wine daily. She was a poor historian, displaying impaired memory/cognition, stating that she was on vacation but unable to provide details regarding when she arrived or how she would return home.

On a general physical exam, she appeared disheveled, slightly pale and malodorous. She spoke with a slight delay and stuttered periodically. She had multiple bruises over her body in various stages of healing. Her neurological exam was significant for ataxia and dysmetria with intention tremor, positive Rhomberg’s test, and positive heel-to-shin test.

**Figure 1:**
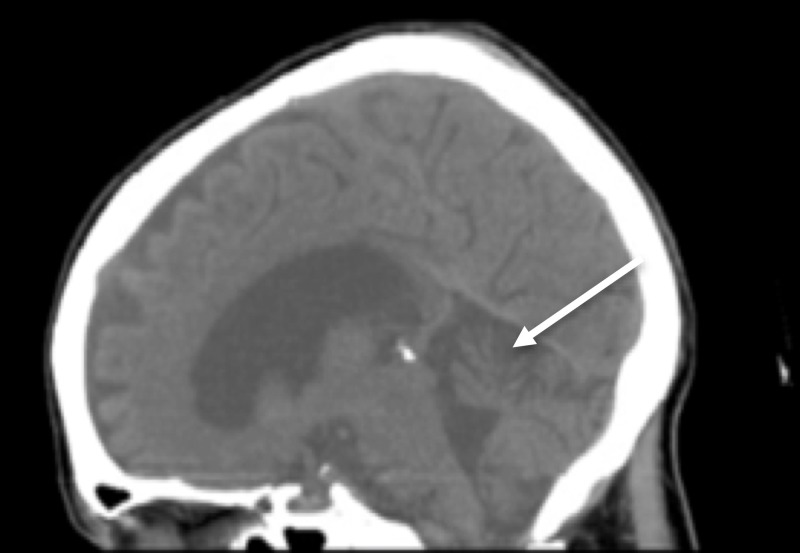
Midsagital CT of the head Midsagital CT of the head shows cerebellar atrophy, most pronounced in the vermis (arrow)

A CT of her brain revealed cerebellar vermian atrophy but was otherwise unremarkable (Figure 1).

**Figure 2:**
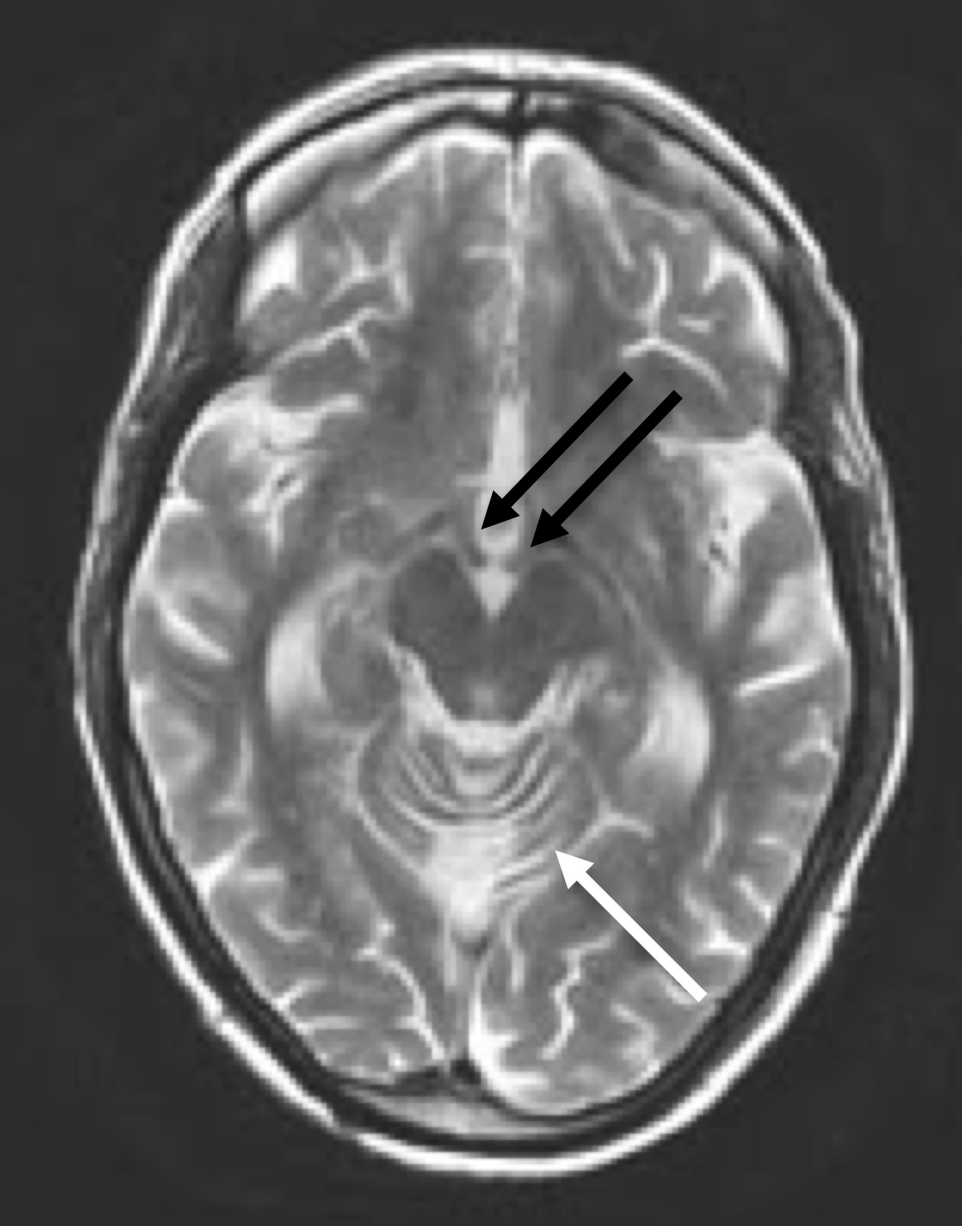
Axial T2-weighted MRI of the head Axial T2-weighted MRI of the head at the level of the midbrain shows prominent vermian folia and abnormal increased signal in the superior vermis (white arrow) as well as normal size and signal intensity of the mamillary bodies (black arrows)

**Figure 3:**
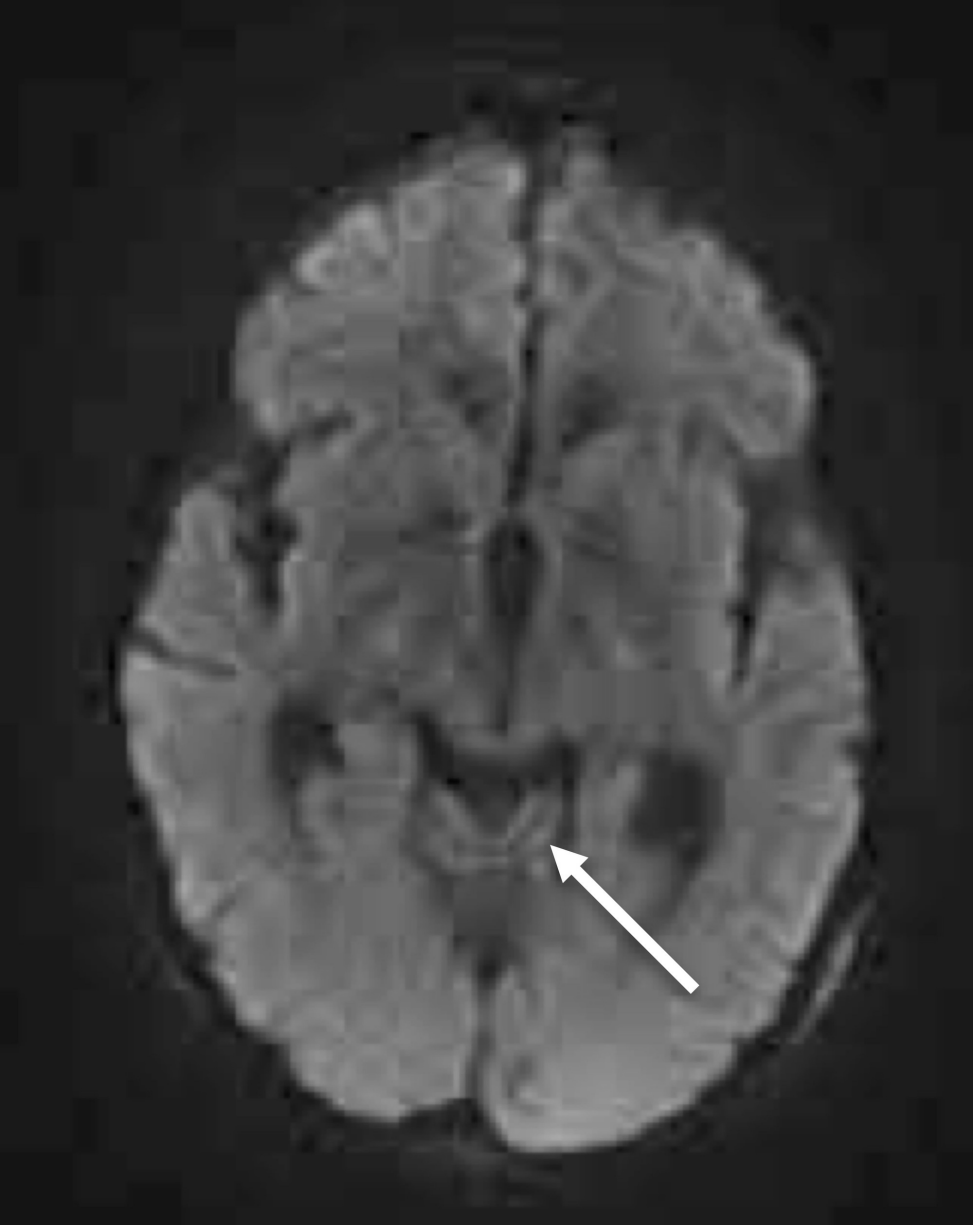
Axial DWI of the head Axial DWI of the head at the level of the cerebellar peduncles shows no restriction in the cerebellar vermis (arrow)

**Figure 4:**
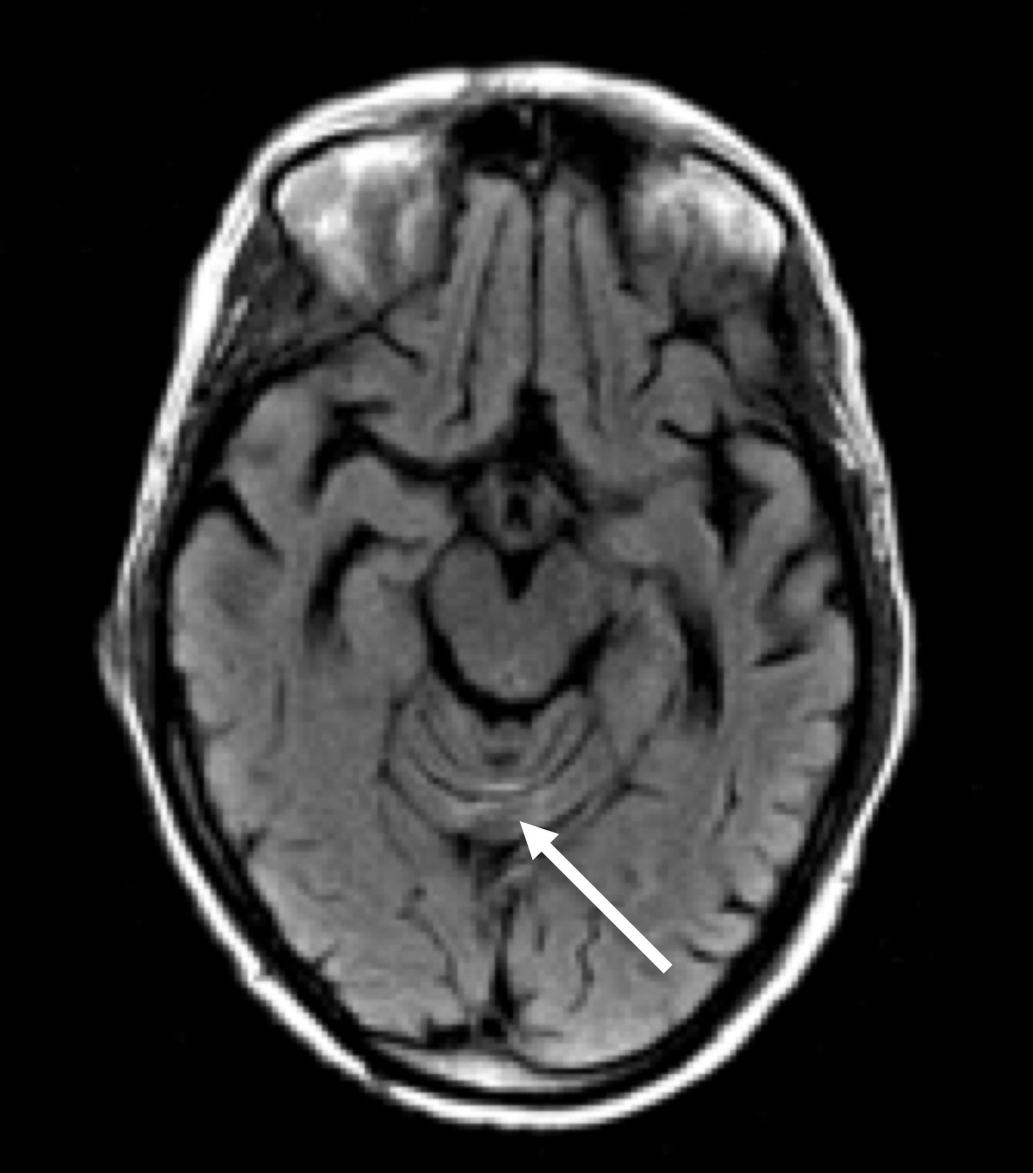
Axial FLAIR MRI of the head Axial FLAIR MRI of the head at the level of the midbrain shows abnormal increased signal in the superior vermis and prominence of vermian folia (arrow)

**Figure 5:**
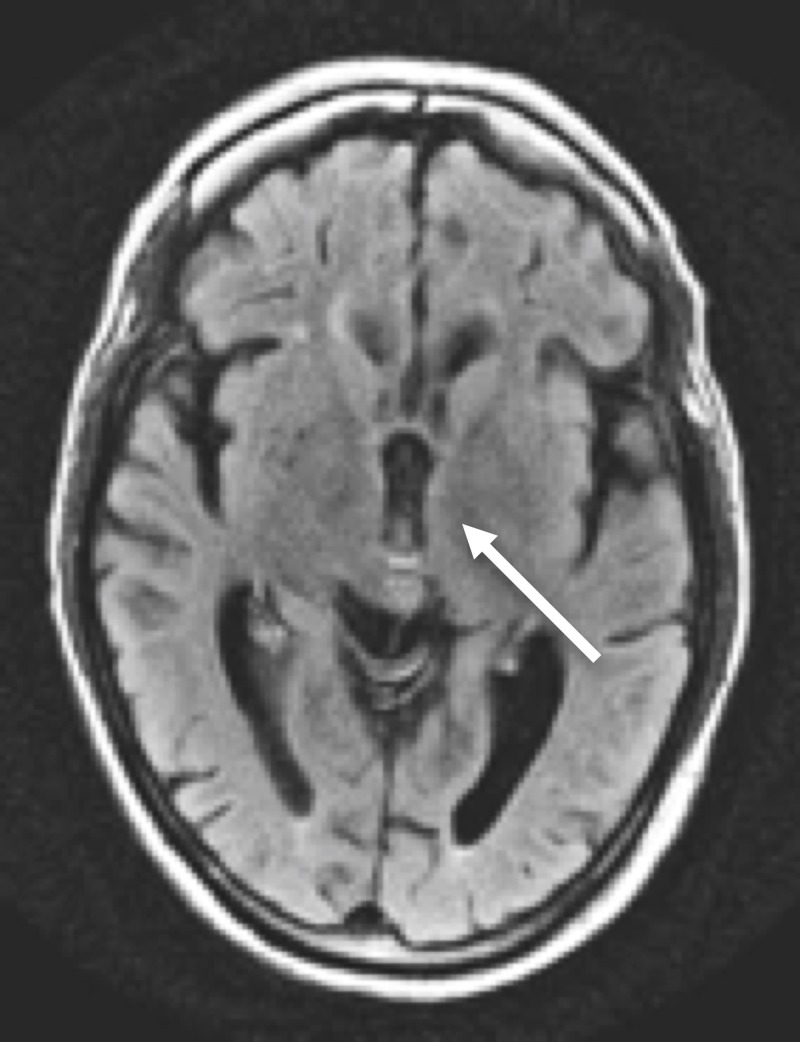
Axial FLAIR MRI of the head Axial FLAIR MRI of the head at the level of the 3^rd^ ventricle shows no significantly increased periventricular signal intensity (arrow)

**Figure 6:**
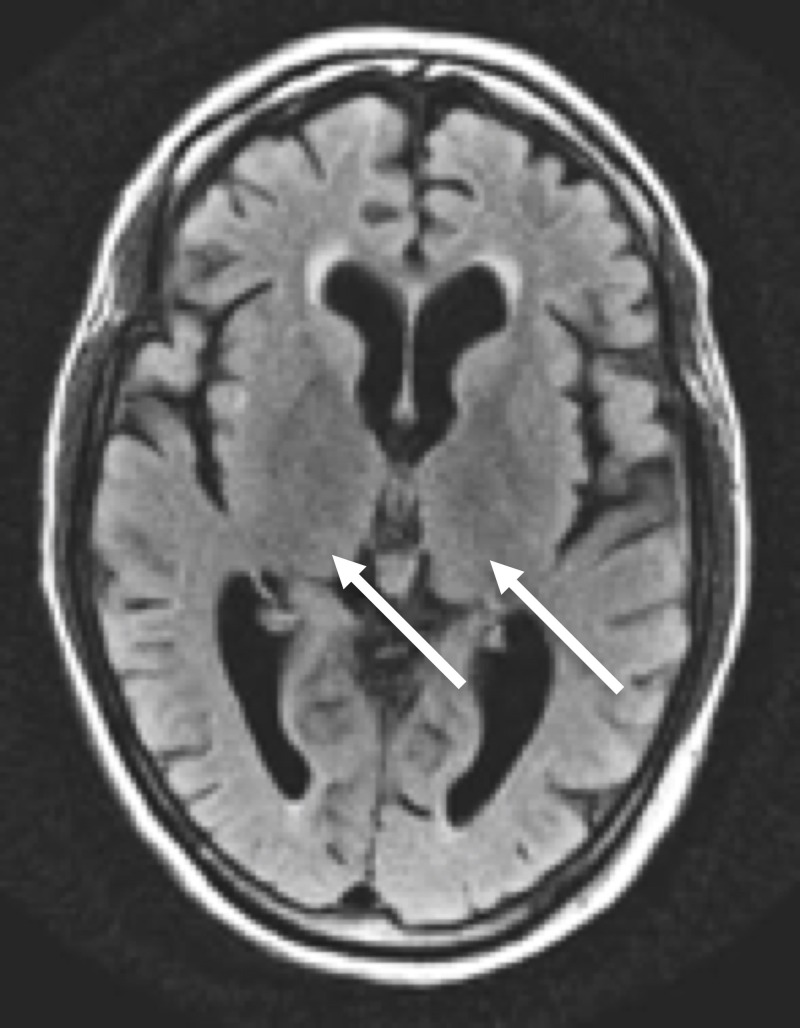
Axial FLAIR MRI of the head Axial FLAIR MRI of the head at the level of the thalami shows no significant increase in thalamic signal intensity (arrows)

**Figure 7:**
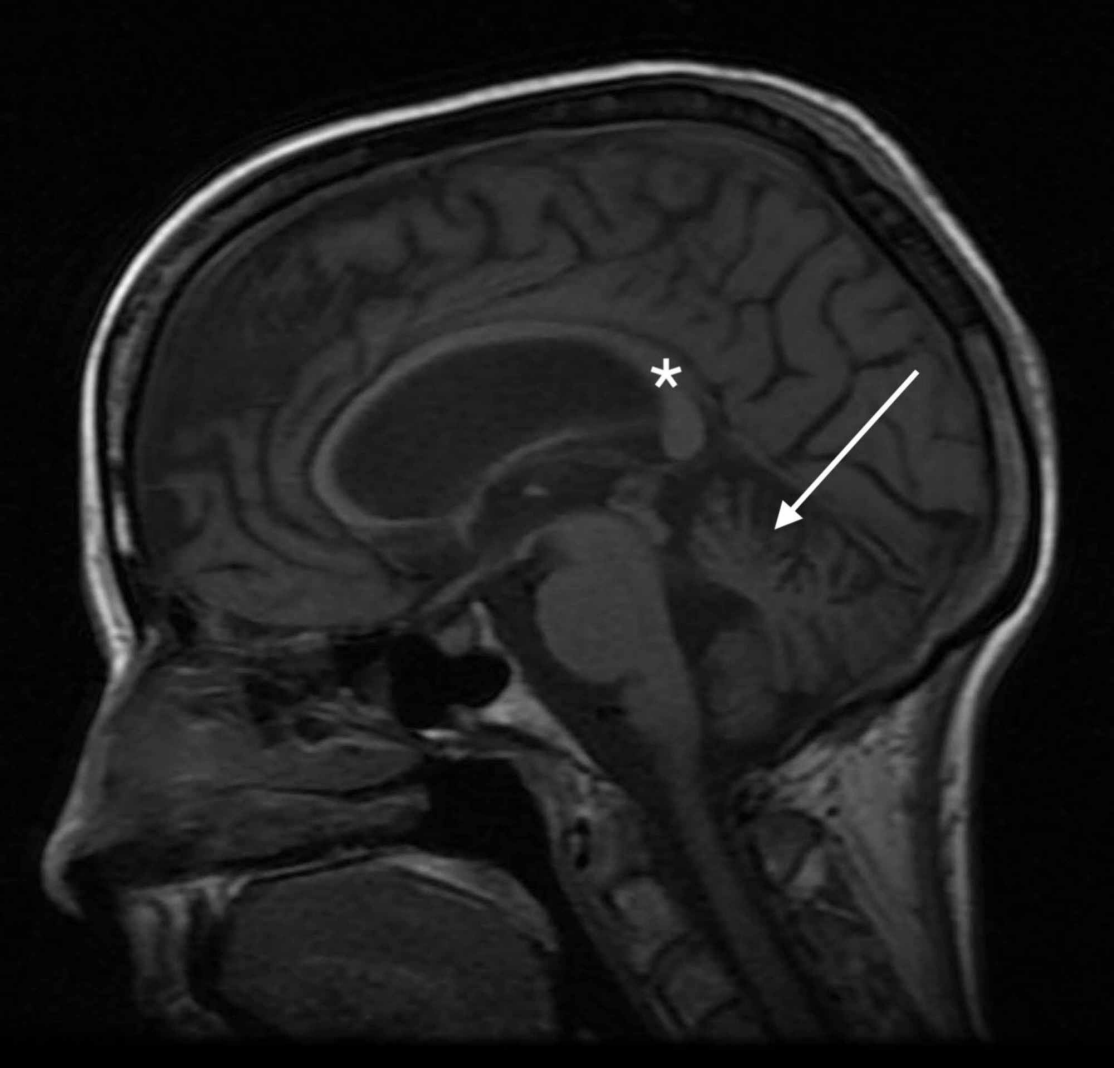
Mid-sagittal T1-weighted MRI of the head Mid-sagittal T1-weighted MRI of the head shows cerebellar atrophy (arrow) and a normal corpus callosum (star)

MRI of her brain demonstrated cerebellar volume loss, increased FLAIR signal in the cerebellar vermis, normal corpus callosum and normal mammillary bodies (Figures 2-7).

The patient’s symptoms dramatically improved following administration of IV thiamine 500 mg.

## Discussion

Wernicke encephalopathy (WE) is a part of Wernicke-Korsakoff syndrome, the neurological complication of thiamine deficiency due to malnutrition from chronic alcoholism, malabsorption, AIDS, anorexia nervosa, and hyperemesis gravidarum among other causes [1-3]. WE is an acute syndrome that necessitates emergent treatment to prevent death or progression to Korsakoff syndrome (KS), the chronic neurologic sequelae stemming from prolonged metabolic dysfunction [2,5]. KS is most often seen in chronic alcohol abusers, primarily characterized by anterograde and retrograde memory deficits with preservation of long-term memory. WE is classically described as a triad of encephalopathy, ophthalmoplegia, and gait ataxia; however, studies involving autopsies with confirmed cases demonstrated that only 1/3 of patients present with the triad, with delirium being the most common symptom [4]. Additional symptoms include hypothermia, peripheral neuropathy, protein-calorie malnutrition, and vestibular dysfunction (with or without hearing loss) [8]. Patients can also present with cardiac conditions including tachycardia, exertional dyspnea, elevated cardiac output and EKG abnormalities, all of which can be reversed with thiamine treatment [4].

Thiamine is an essential B vitamin, with its active form functioning as a co-factor for pyruvate dehydrogenase and alpha-ketoglutarate dehydrogenase [9]. Deficiencies, especially during times of high metabolic demand and high glucose load, produce neuronal changes and structural lesions from a combination of altered neurotransmitter levels, NMDA receptor excitotoxicity, a breakdown of the blood brain barrier, and increased production of free radicals [10]. WE and KS patients exhibit great variability in these structural changes, with atrophy of mammillary bodies in 80% of cases, a highly specific feature of chronic WE and KS [1,5]. 

Although not required for a diagnosis of acute WE, “typical” MRI findings include hyperintense FLAIR/T2 signal in the thalami, mammillary bodies, tectal plate, periphery of the third ventricle, and periaqueductal area [3,6]. Approximately 50% of patients with WE exhibit “atypical" MRI findings including FLAIR/T2 hyperintensity in the superior cerebellum, cranial nerve nuclei, red nuclei, dentate nuclei, caudate nuclei, splenium, and cerebral cortex [6]. Historically, these “typical” findings were thought to be more associated with alcoholics while the “atypical” findings were more associated with non-alcoholics. However, recent literature suggests that “typical” and “atypical” findings are less closely linked to alcohol-related and non-alcohol related WE than previously reported, as in our patient with alcohol-related WE displaying only “atypical” MRI features [7]. Differential diagnosis in patients with MRI findings of WE would include arterial/venous infarction (expected to show restricted diffusion), viral encephalitis (expected to have greater areas of increased FLAIR/T2 signal; atrophy would be unusual) and Marchiafava-Bignami Disease (expected to have callosal signal abnormality).

A diagnosis of WE is made based on the clinical presentation. A delayed or missed diagnosis occurs more frequently in non-alcoholic patients, especially if the classic triad is absent [4,8]. To avoid such diagnostic errors, WE can be identified in patients who have two or more Caine criteria: dietary deficiency, oculomotor abnormalities, cerebellar dysfunction, and altered mental status/mild memory impairment [11]. When compared to the classic triad, the Caine criteria increased sensitivity from 22% to 85% in non-alcoholic patients [11]. Currently there is no laboratory test to specifically diagnose WE. However, a 25% increase of Erythrocyte thiamine transketolase can help establish the diagnosis of WE, especially if levels normalize with clinical improvement [12]. Pyruvate and lactate levels may also be elevated due to impaired aerobic metabolism due to impaired Krebs cycle enzymatic activity [9]. Previously mentioned MRI abnormalities, specifically T2 signal abnormalities, can disappear within 48 hours of treatment; however some of the clinical symptoms may not fully resolve [4,13].

Treatment for WE rests entirely on prompt thiamine supplementation. As IV thiamine is safe, inexpensive, and effective, empiric treatment prior to acquiring labs is highly recommended [14]. The prescribed regimen is 500 mg IV over 30 min, every 8 hours daily for two consecutive days and 250 mg IV or IM daily for an additional five days in addition to other B vitamins [15]. Oral administration is discouraged because thiamine absorption in alcoholic or malnourished patients is unreliable [16]. A clinical pearl for prevention is to ensure administration of thiamine prior to glucose in all cases [15].

## Conclusions

Wernicke encephalopathy is a serious medical condition resulting from thiamine deficiency. Consequences of delayed treatment are dire, making prompt diagnosis and management critical. Although the diagnosis is clinical, laboratory and MRI findings may help support a diagnosis and can establish response to therapy. Prompt parenteral thiamine supplementation is recommended in patients who meet the Caine criteria, prior to administering glucose.
